# Ozonation of three different fungal conidia associated with apple disease: Importance of spore surface and membrane phospholipid oxidation

**DOI:** 10.1002/fsn3.1618

**Published:** 2020-08-19

**Authors:** Marielle Pagès, Didier Kleiber, Frédéric Violleau

**Affiliations:** ^1^ Equipe Physiologie Pathologie et Génétique Végétales (PPGV) Institut Polytechnique de Toulouse Université de Toulouse Toulouse Cedex 03 France; ^2^ Laboratoire de Chimie Agro‐industrielle (LCA) INRA, INPT, INP‐EI PURPAN Université de Toulouse Toulouse France

## Abstract

Although ozone (O_3_) is a well‐known bactericide and fungicide, the required dose of ozone can depend significantly on the targeted pathogens. The present research evaluates the variation in sensibility to ozone of three fungal species from a single fungal group. The three fungal species selected, *Venturia inaequalis*, *Botrytis cinerea*, and *Neofabreae alba*, belong to the Ascomycota group and attack apples. The fungi were exposed to ozone by bubbling directly into the spore solutions (treatment period ranged from 0.5 to 4 min, ozone concentration in inlet gas ranged from 1 to 30 g/m^3^). The rates of germination were determined, and the level of peroxidation of the lipid membrane was quantified based on the malondialdehyde (MDA) production. The results indicate that ozone effectively reduces spore development and suggest that the fungi differ in sensitivity. To reduce by 50% the spore germination rate of *N. alba*, *B. cinerea*, and *V. inaequalis* requires ozone doses of 0.01, 0.03, and 0.07 mg/ml, respectively. Spore sensitivity seems to be directly linked to spore surface. For all the fungal species, the MDA level and the level of spore inactivation both increase with ozone dose, which confirms that ozone alters the cell membrane.

## Graphical abstract

Although ozone (O_3_) is a well‐known bactericide and fungicide, the required dose of ozone can depend significantly on the targeted pathogens. Thanks our results, spore sensitivity seems to be directly linked to spore surface. For all the fungal species, the MDA level and the level of spore inactivation both increase with ozone dose, which confirms that ozone alters the cell membrane.

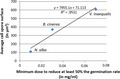

## 1 INTRODUCTION

Ozone is a promising alternative to limit microbiological pressure on fruits and vegetables. Ozone gas or ozone dissolved into water (called “ozonated water” for convenience) inactivates bacteria, fungi, and viruses (Guzel‐Seydim, Greene, & Seydim, 2004) and leaves a low remanence in the environment compared with conventional disinfectants, which is one of its main advantages. The half‐life of ozone dissolved in water at 20°C is between 20 and 30 min (Khadre & Yousef, 2001), and it decomposes mainly into nontoxic products, such as oxygen (Sharpe et al., 2009). Currently, this strong oxidant is used to disinfect drinking water, industrial wastewater, and food‐processing equipment.

Although numerous experiments have been conducted to verify the inactivation of micro‐organisms by ozone, few reports state the dose required to kill a specific species. The efficiency of ozone depends on how it is applied (ozone gas vs. ozonated water), the interaction between ozone and the fruit support, and the quantity, type, and development stage of the micro‐organism.

For example, bacteria are more sensitive than fungi. Moore, Griffith, and Peters (2000) made a comparative study between bacteria and yeasts. Applying ozone gas for one hour at 2.0 ppm reduces the viability of *E. coli* by more than 6.0 Log. Under the same conditions, the viability of the yeast *Rhodotorula rubra* is reduced by 0.57 Log. Moore et al. conclude that the thin cellular wall of yeast (generally 100.0–200.0 nm thick) may protect cells by hindering ozone penetration until active sites of the plasmic membrane. Likewise, Restaino, Frampton, and Hemphill (1995) showed that, under certain treatment conditions, ozonated water kills 4.5 Log of the cells of the two yeast species *Candida albicans* and *Zygosaccharomyces bailii*. The same treatment inhibits <1.0 Log of *Aspergillus niger* spores, even after 5 min of exposure. Finally, Palou, Smilanick, Crisosto, and Mansour (2001) demonstrated that, after five days of incubation, the radial growth of Penicillium italicum is significantly reduced by continuous exposure to ozone (0.3 ppm at 5°C for 4 days). Conversely, *Penicillium digitatum* is insensitive to the same treatment. This difference is attributed to the variability in spore morphology.

The capacity of ozone to destroy micro‐organisms is influenced by the wall composition. Ozone inactivates its biological targets by progressive oxidation of the vital cellular compounds (Guzel‐Seydim et al., 2004), with the cell surface being the primary target (Komanapalli & Lau, 1996; Makky, Park, Choi, Cho, & Kim, 2011; Scott & Lesher, 1962). First, ozone attacks polyunsaturated fatty acids, which leads to a modification of the membrane and initiates a chain reaction that transforms polyunsaturated fatty acids (Iriti & Faoro, 2008) into malondialdehyde [MDA, CH₂(CHO)₂]. This lipid peroxidation by ozone has been described for bacteria (Cho, Kim, Kim, Yoon, & Kim, 2010), micro‐alga (Wu, You, Zhang, Chen, & Lee, 2011), and fungi (Pagès, Kleiber, Pierron, & Violleau, 2015).

In this work, we compare how ozone affects the three species *V. inaequalis*, *B. cinerea*, and *N. alba*, which belong to the fungal group Ascomycota and attack the same fruit target. The aim was to elucidate whether the sensibilities of these species differ by monitoring the rate of inactivation of germination. By measuring the level of lipid peroxidation, we also clarify whether the inactivation rate can be explained by the variation in reactivity of the cell membrane.

## 2 MATERIAL AND METHODS

### 2.1 Cultures and conidium suspensions

*Venturia inaequalis* was isolated on apple and cultivated on Malt Extract Agar. Petri dishes were incubated at 17°C (lighting 16 hr/day) for at least 7 days before conidium sampling. For the *Neofabreae alba* conidia, contaminated apples (provided by Centre Expérimental des Fruits et Légumes) were first stored for 6–8 months in a cold chamber (4°C) to obtain mother structures containing conidia. Next, these mother structures were placed in sterile water before being crushed to release spores. *Botrytis cinerea* (CBS 126.58) was cultivated at 21°C on Potato Dextrose Agar, and aqueous suspensions were made with three‐week‐old cultures.

For all species, water was added to adjust the concentration of spores in suspension to about 1.0 × 10^5^ spores/ml as per a count using a Malassez cell.

### 2.2 Ozone system

Ozone was produced by using a LAB2B Ozone Generator (Ozonia, Dubendorf, Switzerland) supplied with oxygen by an oxygen bottle for concentrations exceeding 10 g/m^3^ and a nitrogen–oxygen mixture (78.09%, 20.94% vol.) for concentrations of 1 or 2 g/m^3^. The inlet ozone concentration was controlled by using an ozone analyzer BMT 961 (BMT). An ozone bubble column was used to bubble ozone gas in 10 ml conidium aqueous suspensions to obtain a constant concentration of ozone dissolved in the water (Pagès et al., 2015). The gas flow rate was fixed at 10 L/hr.

The ozone concentrations in the inlet gas were 1, 2, 10, 20, and 30 g/m^3^, and the bubbling continued for 0.5, 2.0, 4.0, and 5.0 min. The dose of ozone applied to the solution of spores may be estimated by using

$$\frac{{\left[O_{3}\right]}_{g}\times t\times D}{60\times V}=\text{applied ozone dose,}$$

where [O_3_]_g_ is the ozone concentration in inlet gas in g/m^3^, *t* is the duration of ozone application in minutes, *D* is the flow rate in L/hr, and *V* is the volume of the solution in ml (which is equal to 10 ml). The dose is expressed in mg/ml.

### 2.3 Evaluation of germination rate

After ozonation, Malassez cells were used to determine the germination rate. Twenty microliters of conidium aqueous suspensions was placed on each Malassez cell, following which the cells were placed in a high‐humidity compartment (at least 90% relative humidity). This compartment was maintained in culture chamber under conditions favorable for the development of the targeted fungus (Table 1).

**TABLE 1:** Culture conditions for *Venturia inaequalis, Botrytis cinerea*, and *Neofabreae alba* after deposition on Malassez cell

Fungus	*V. inaequalis*	*B. cinerea*	*N. alba*
Temperature (°C)	17	21	23
Incubation period (hr)	24	48	24

After this period, 100 spores were chosen at random and the germinated spores in this sample were counted by using an optical microscope (Leica DM750 light microscope, Leica Microsystems GmbH). Table 2 presents the counting criteria for each species. Four counts were made by treatment, and each treatment was repeated twice.

**TABLE 2:** Decision criteria for measuring germination rate of *Venturia inaequalis, Botrytis cinerea*, and *Neofabreae alba* (INRA, and personal communication)

Fungus	*V. inaequalis*	*B. cinerea*	*N. alba*
Spore length (in µm)	12–22	6–18	15–18
Spore width (in µm)	6–12	4–11	1–3
Length of germinal tube of germinated spores	When germinal tube is twice as long as nongerminated spore	When germinal tube is longer than 3 µm	When length of germinal tube is greater than or equal to half the spore length

### 2.4 Lipid peroxidation

After bubbling ozone into the spore solutions, the solutions were concentrated by centrifugation to a concentration of 1.2 × 10⁶ spores/ml. Each sample was sonicated for 5 s (VibraCell 75185, Sonics and Materials). The quantity of MDA produced was determined by using a OXltek TBARS Assay Kit (Enzo, ALX‐850‐287). Briefly, after adding SDS and the TBA/buffer reagent, the samples were incubated at 95°C for 60 min and then cooled to room temperature. The samples were then centrifuged, and the supernatant was removed for analysis. The absorbance was measured at 532 nm (spectrophotometer Sunrise, Tecan) and calibrated by using a MDA standard curve. The concentration of MDA is expressed in nmol/ml.

### 2.5 Data analyses

The statistics were analyzed by using the software XLSTAT. First, a Shapiro–Wilk test was applied to verify a normal distribution. If normality conditions were met, the data variance was analyzed. The different treatments were compared with the control with the matching test of Newman–Keuls.

If normality conditions were not met, we verified that the standardized residuals fell between −3 and 3. Where appropriate, the data were subjected to an analysis of variance, which is considered a sufficiently robust test. The error tolerance *α* was set at 0.05.

## 3 RESULTS AND DISCUSSION

### 3.1 Inactivation of Ascomycota spores

We tested the inhibition of germination due to exposure to ozone through bubbling in conidial aqueous suspensions of *B. cinerea*, *V. inaequalis*, and *N. alba*. Different treatments (i.e., application time and ozone concentration) were used to study the effect of ozone exposure.

**FIGURE 1:**
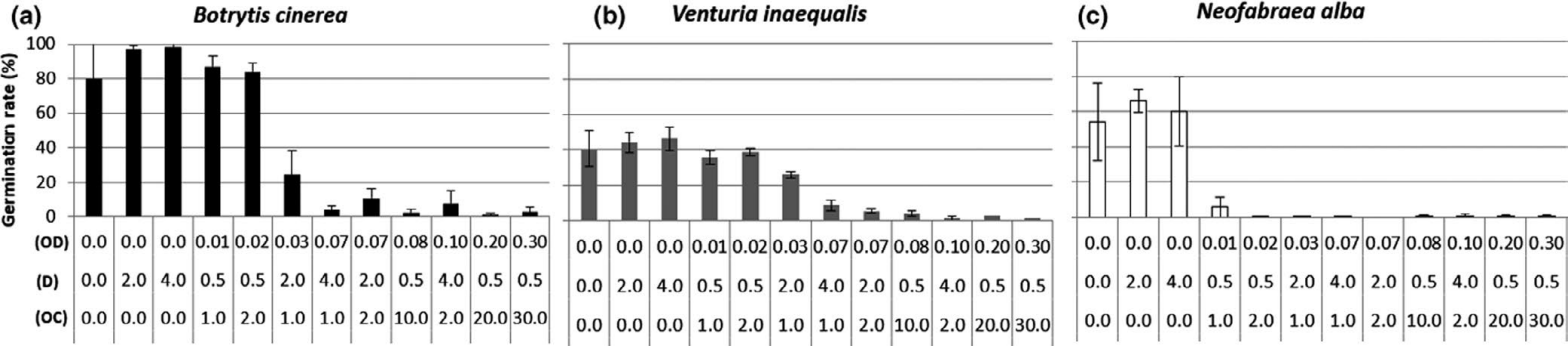
Effect of ozone on the germination rate of (a) *Botrytis cinerea*, (b) *Venturia inaequalis*, and (c) *Neofabreae alba*. Equivalent treatments are designated by the same letter (Newman–Keuls tests, *α* = 0.05). OD = ozone dose (mg/ml); *D* = duration of bubbling (min); OC = ozone concentration (g/m^3^)

Figure 1 shows that bubbling with nitrogen–oxygen mixture without ozone for 2.0–4.0 min causes no reduction in germination, which means that the presence of ozone, as opposed to the mechanical action of bubbling, is what limits fungal growth, regardless of the micro‐organism considered. In some cases, the bubbling of nitrogen–oxygen mixture air appears to favor the germination rate (i.e., for *B. cinerea* and *N. alba*). Presumably, the agitation or oxygenation caused by bubbling favors the germination dynamics.

Figure 1a–c shows that the measured germination rate of the conidia of the three fungal species decreases as the ozone dose increases. When the ozone dose exceeds 0.07 mg/ml, the germination rates of *B. cinerea*, *V. inaequalis*, and *N. alba* are similar and close to 0% (i.e., <2.6%, 3.8%, and 0.5%, respectively). These germination rates differ statistically from the control values, which are 80.0%, 40.5%, and 54.2%, respectively.

Ozone doses between 0.01 and 0.07 mg/ml lead to differences in the sensitivity of the fungi, namely, *B. cinerea* and *V. *inaequalis become less sensitive than *N. alba*. In fact, a dose of 0.01 mg/ml suffices to limit the germination rate of *N. alba* to 5.8%. For the other two fungal species, this dose does not significantly reduce the number of germinated spores compared with the control values. Regarding *B. cinerea* and *V. inaequalis*, a dose of 0.03 mg/ml is required to obtain germination rates significantly less than the control values. An ozone dose of 0.07 mg/ml is required to limit germination rates below 10%.

These initial results confirm that bubbling ozone through the spore solutions inhibits spore germination of *B. cinerea*, *V. inaequalis*, and *N. alba*. In addition, these results show that sensitivity to ozone depends on the fungal species.

*Neofabreae alba* is the most sensitive to ozone of the three fungal species studied herein, which can be attributed to variations in the cell‐surface composition (Alexopoulos et al., 2013) and/or to the different defense mechanism used to combat oxidative stress (Antony‐Babu & Singleton, 2009) due to sensor kinases sensibility, transmitting oxidative stress signal efficiency, scavenging systems reactivity, …. Moreover, in addition, differences in size and morphology of the spores of the three species may also contribute to the different sensitivities to ozone.

**FIGURE 2:**
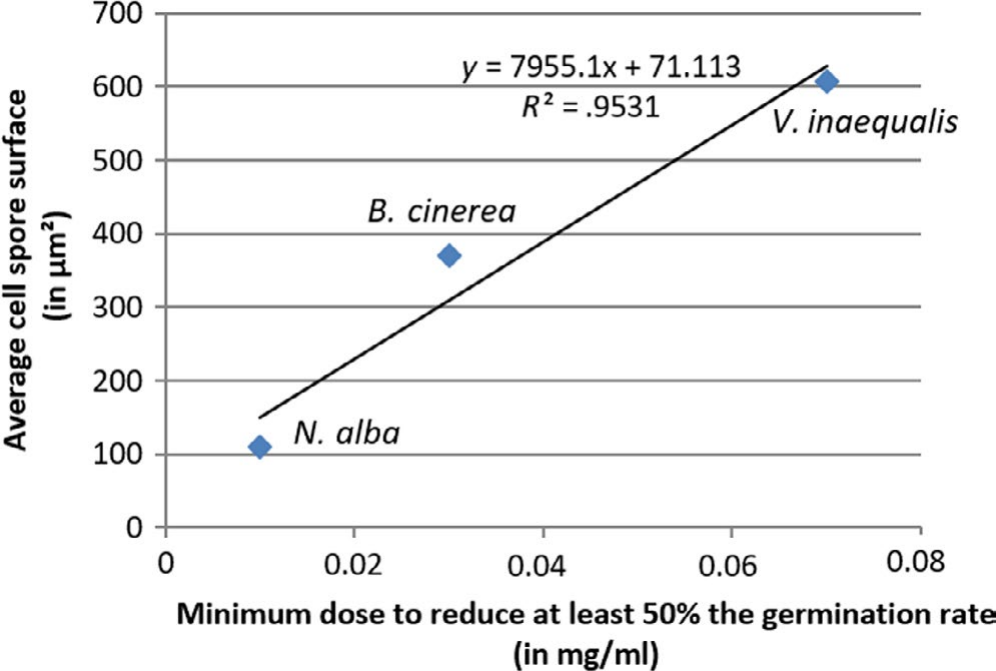
Minimum dose to reduce germination rate by at least 50% based on the average cell spore surface of each fungal species

Figure 2 shows that, for similar spore quantities, the dose required to inactivate *N. alba's* spores is lower than for the two other species: A dose of 0.01 mg/ml is required to reduce by 50% the spore germination rate of *N. alba*, whereas *B. cinerea* and *V. inaequalis* spores require a dose of 0.03 and 0.07 mg/ml, respectively. The conidia of *N. alba* are particularly long and narrow: The global cell surface of this conidium estimated from the calculated average length and width (Table 2) is about three‐ to fivefold less than that of the conidia of *B. cinerea* and *V. inaequalis*. The coefficient of determination (*R* ^2^) of the regression is .9531. The linear model explains 95.31% of the variability of the points. Therefore, it is possible to affirm, that in addition of biochemical pathways and gene expression specificities which can be fungal species‐dependent, the surface cell parameter, and thus, the total area available for oxidation by ozone seems to be an interesting factors to determine the required ozone dose.

Moreover, the difference dose requirements could be explained by the nature of the spores. In this experiment, the solution contained about 100,000 spores/ml. Normalizing the minimum dose required to reduce by at least 50%, the germination rate to the total surface of the cell spores gives a required ozone dose of 9.10 × 10^−10^, 8.09 × 10^−10^, and 1.15 × 10^−9^ mg/µm^2^ per unit surface to kill the micro‐organisms *N. alba*, *B. cinerea*, and *V. inaequalis*, respectively. We thus consider that no significant difference exists between these data. Therefore, the nature of the membrane or cell wall seems not to be a main factor for determining the sensibility of these fungi to ozone.

The inactivation of micro‐organisms by ozone is a complex process. Acting by contact, ozone can oxidize many cell components. For some researchers, ozone itself is the main inactivator, whereas others believe that it is the ozone decomposition by‐products (°OH, °O_2_‐, and HO_3_°) that inactivate the micro‐organisms (Khadre, Yousef, & Kim, 2001).

Most researchers agree that the first victim of ozone is the cell surface, which is corroborated by the fact that ozone is estimated to be too reactive to deeply penetrate tissue without altering the cell membrane. Thus, only a small amount of ozone passes through the membrane (Pryor, 1992). The first step in this process is the modification of the membrane permeability (Miller, Silva, & Brandão, 2013), which involves the oxidation of the phospholipids that constitute the cell membrane. By reacting with polyunsaturated fatty acids, ozone causes peroxidation, which affects the membrane fluidity. This "ozonolysis" reaction cleaves the double bonds of alkenes in polyunsaturated chains, creating ozonides (Criegee mechanism). These ozonides are then broken down into organic radicals, peroxides, and aldehydes (Iriti & Faoro, 2008).

Given the hypothesis that the first target of ozone is the phospholipid membrane, the second part of this study compares the level of phospholipid peroxidation between the three fungal species.

### 3.2 Comparison of lipid peroxidation level

**FIGURE 3:**
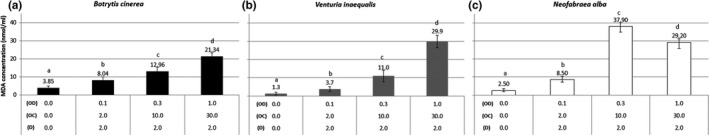
MDA concentrations (in nmol/ml) as a function of ozone dose applied by bubbling in spore solutions of (a) *Botrytis cinerea*, (b) *Venturia inaequalis*, and (c) *Neofabreae alba*. The terms are grouped based on the results of variance analysis. Each group is labeled by a letter (a–d). OD = ozone dose, mg/ml; *D* = duration of Bubbling (minutes); OC = ozone concentration in incoming gas (g/m^3^)

The mechanism or mechanisms through which ozone affects fungal species remain unclear, and few studies have focused on fungal spores. Based on the data obtained from other models such as bacteria, a possible mechanism to consider is the deterioration of the cell surface of the spore, which includes peroxidation of the membrane phospholipids due to contact with ozone. To investigate this, we quantify the MDA that results from oxidation of the phospholipids. Figure 3 shows the MDA concentration as a function of ozone dose applied by bubbling in spore suspensions of *B. cinerea*, *V. inaequalis*, and *N. alba*.

The MDA concentration gradually increases with increasing ozone dose. For *B. cinerea*, ozonation with 30.0 g/m^3^ for two minutes significantly increases the MDA concentration compared with the spore suspension control samples (from 3.85 to 21.34 nmol/ml). Similarly, control samples of *V. inaequalis* have an average MDA concentration of 1.30 nmol/ml, whereas the most ozonated samples have an average MDA concentration of 29.85 nmol/mL. These results are consistent with published results. For example, Wu et al. (2011) showed that increasing the dose of ozone to *Amphidinium sp*. (seaweed) results in an increased MDA concentration.

For *N. alba*, bubbling ozone leads to a significant production of MDA. However, the concentration decreases from 37.9 to 29.2 nmol/mL in going from treatment 3 to 4. Treatment 4, with an application of 30 g/m^3^ of ozone for 2 min, may attack the MDA released by the peroxidation of phospholipids. Cho et al. (2010) also recorded a peak of MDA concentration from 0.75 to 1 Log inactivation on bacteria. They attributed this peak to the ozone attacking the newly produced MDA molecules. Higher ozone doses lead to membrane lysis and the release of cellular compounds, which may become new targets for the ozone. Such a mechanism would explain this new increase in MDA production.

**FIGURE 4:**
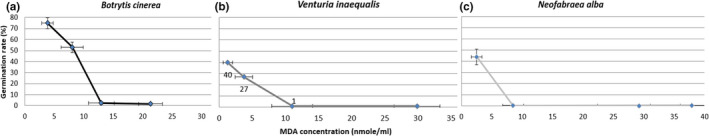
Germination Rate (in %) of the spores of (a) *Botrytis cinerea*, (b) *Venturia inaequalis* and (c) *Neofabreae alba* according to the MDA concentration (in nmol/ml of sample)

We now turn to the question of whether the loss of functionality of the membrane, as demonstrated by the peroxidation of phospholipids, can explain spore inactivation. The level of phospholipid oxidation is linked to the germination rate (Figure 4).

The nonozonated control samples, which are still able to grow, correspond to the lowest MDA levels of 3.85, 1.3, and 2.5 nmol/ml for *B. cinerea*, *V. inaequalis*, and *N. alba*, respectively. In contrast, as the MDA concentration increases, the germination rate decreases. Increasing the amount of MDA produced correlates directly with spore inactivation. These results confirm those of Cho et al. (2010) which show that increasing MDA production correlates with increased *E. coli* inactivation. MDA production is connected to the deterioration of the cell membrane, leading to the loss of respiratory activity and ultimately to cell death.

For the three species studied in the present work, MDA production continues even when spores are unable to grow: Reactions between ozone (or free radicals) and spore components persist, even when the lethal stage is attained. Note that ozonation does not result simply in oxidative stress causing the implementation of defenses that generate MDA. If this were the case, MDA production would stop once the cell dies. In fact, the oxidative chemical reactions between ozone and phospholipids lead to the formation of MDA, so the formation of MDA is not just a biological response. However, the hypothesis of oxidative stress and a spore response prior to its death cannot be completely ruled out. Ong and Ali (2015) recently demonstrated that, in response to an increasing dose of ozone, more cells synthesize reactive oxygen species (ROSs). These ROSs come from the mitochondrial respiratory chain, which is a major source of endogenous ROSs, especially when mitochondria are damaged. However, the counts show that all spores were unviable. Ong and Ali concluded that the accumulation of ROSs caused oxidative damage and cell death. The alteration of the cell surface could be a first step but not the only step involved in cell inactivation.

## 4 CONCLUSION

The aim of this study was to understand the different sensitivities to ozone of three fungal species. Although ozone inactivates all three Ascomycota fungal spores considered in this study, the sensitivity to ozone varies with the species as a function of spore size and morphology. Both MDA production and the level of spore inactivation increase with ozone dose.

Ozone initially reacts primarily with the surface of organic cells via the Criegee mechanism, leading to the production of malondialdehyde (MDA). MDA is produced in particular by the action of ozone on polyunsaturated membrane phospholipids, and its quantification highlights the degree of peroxidation of membrane phospholipids. For the three fungal species studied, the MDA concentration increases, starting at the lowest dose of ozone. Thus, ozone oxidizes the membrane phospholipids. The alteration of phospholipids, one of the major constituents of the plasma membrane, results in the embrittlement of the cell surface and leads to its fluidity. The present results show the possibility and the need to accurately determine the required dose.

Although deploying ozone against micro‐organisms has always been efficient, preliminary studies based on simple systems such as the ozonated bubble column used herein are required to determine the sensitivity to ozone of the biological target.

## CONFLICT OF INTEREST

Declare any conflicts of interest or state that there are none to declare.
